# Acknowledgement to Reviewers of *Plants* in 2013

**DOI:** 10.3390/plants3010141

**Published:** 2014-02-26

**Authors:** PlantsEditorial Office

**Affiliations:** MDPI AG, Klybeckstrasse 64, CH-4057 Basel, Switzerland

The editors of *Plants* would like to express their sincere gratitude to the following reviewers for assessing manuscripts in 2013:
Abella, ScottKouretas, KouretasRomeis, TinaAnderegg, WrlKrajňáková, JanaRoy, MimiAnwar, RaheelKramer, Elena M.Rozwadowski, KevinBaluska, FrantisekKreft, JürgenSasaki-Sekimoto, YukoBarot, SébastienKrizek, BethSawa, ShinichiroBrandão, R. L.Kuboyama, TsutomuScherer, GüntherBrière, ChristianKutschera, UlrichSchippers, JosBui, E.n.Lamb, RebeccaSchneider, AnjaBykova, OlgaLamport, Derek T. A.Schreiber, StefanCarter, Clay J.Landis, J BSeifert, Georg J.Causier, BarryLanglois, EstelleShimada, YukihisaChatterjee, MithuLapointe, LineShimmen, TeruoCorreia, P.j.Larsen, Paul B.Skaltsa, Helen D.Delourme, RégineLaufs, PatrickSpringer, Patricia S.Diekmann, KerstinLee, Bao-ShiangSreelatha, S.Distefano, GaetanoLee, Jeong-DongStacey, GaryDolferus, RudyLi, JinyunStirk, Wendy A.Dresselhaus, ThomasLi, LeiStotz, Henrik U.Emery, Sarah M.Lindberg, SylviaTakeda, SeijiFasoula, VasiliaLo, Yi-chenTaylor, MackenzieFeijó, José A.Lora, J.Teeri, TeemuFinet, CédricLüttge, UlrichTeige, MarkusGandin, AnthonyLuu, Doan-trungTill-Bottraud, IrèneGeitmann, AnjaMa, WeiTominaga, MGilliham, MatthewMarin, JuanToribio, MarianoGlenda, GillaspyMarshall, Diane L.Toriyama, KinyaGuadagno, CarmelaMasuda, ShinjiTraas, JanHaling, RebeccaMattoo, AutarTzakos, Andreas G.Hamilton, BillMaurel, ChristopheUeoka-Nakanishi, H.Hamilton, Cyd E.Mccormick, SheilaUllrich, CorneliaHedhly, AfifMccubbin, Andrew G.Ushijima, KoichiroHeijmans, KlaasMiller, Penny A.Vandenbussche, MichielHibara, Ken-IchiroMojtahedi, NargesVenkateshwaran, M.Higashitani, AtsushiMontgomery, BerondaViaene, TomHippler, MichaelMorris, RichardVitória, Angela P.Honys, DavidMueller-Roeber, BerndVlachonasios, K.E.Horiguchi, GorouNakano, DaisukeWang, Hui-Min DavidHuang, Wen-LiiNicotra, AdrienneWasternack, C.Hudson, AndrewNonis, AlbertoWelti, RuthIchitani, KatsuyukiPacifico, SeverinaWilliams, Joseph HInzé, DirkParish, Roger W.Winship, Lawrence J.Iwano, MegumiPaschke, ReinhardWu, HanwenJäger, AnnaPautot, VéroniqueYabuta, YukinoriJauh, Guang-yuhPelloux, JérômeYamamoto, TJeong, Soon-ChunPogson, Barry J.Yang, ZhenbiaoJohnson, Norman A.Quesada, VíctorYoshimura, KazuyaKanaoka, MasahiroRaman, HarshYu, JingquanKawagishi-Kobayashi, MakikoRashotte, Aaron M.Zhang, DabingKim, No SooRaven, John A.Zhang, PengKimura, SeisukeRingli, ChristophZhang, XianshengKöhler, ClaudiaRobertson, DominiqueZupkó, IstvánKost, BenediktRoesch-Ely, M.


